# Comparison of a wearable core temperature sensor with rectal thermometry during high-intensity interval running in pregnant and non-pregnant women

**DOI:** 10.1016/j.jesf.2026.200529

**Published:** 2026-10-01

**Authors:** Sofia Brevik-Persson, Jørgen Melau, Emilie Mass Dalhaug, Christina Gjestvang, Lene A.H. Haakstad

**Affiliations:** a Department of Sports Medicine, Norwegian School of Sports Sciences, Norway; b Medical Service Institute, Norwegian Armed Forces, Norway

**Keywords:** Athletes, High-intensity exercise, Temperature, Thermoregulation, Pregnancy, Running

## Abstract

**Background:**

An increasing number of pregnant women, including elite athletes, continue vigorous- and high-intensity exercise during pregnancy. Current pregnancy exercise guidelines primarily recommend at least 150 min of moderate-intensity physical activity per week and provide limited specific guidance on vigorous- and high-intensity exercise in highly trained pregnant women. High-intensity exercise can elevate core temperature (Tcore), raising concerns about maternal hyperthermia and potential fetal risks (e.g. neural tube defects, preterm birth, fetal growth restriction). Accurate monitoring of maternal Tcore may be necessary to evaluate safety. However, pregnancy-related cardiovascular and thermoregulatory adaptations, such as changes in skin blood flow and heat dissipation, may affect temperature estimates, yet few non-invasive and user-friendly methods combine accuracy with practicality during exercise. This study compared the non-invasive CALERA Research Sensor (CRS) with a rectal probe, the gold standard for Tcore measurement, during high-intensity interval running in pregnant and non-pregnant participants.

**Methods:**

Thirty elite and recreational athletes participated, including 15 pregnant participants (31 ± 4 years) and 15 non-pregnant participants (30 ± 6 years). All completed a separate 10-min warm-up, followed by up to five 5-min high-intensity treadmill-intervals separated by recovery periods. Testing was conducted under environmental conditions of 20-21°C and 38-61% relative humidity. Analyses included the intervals, corresponding pauses, and 20 min of post-exercise, during which no cool-down was performed. Agreement was assessed using Bland–Altman plots, with a pre-defined ±0.3°C acceptability threshold. Differences were analyzed using paired *t*-test.

**Results:**

Nearly half of the measurements exceeded the predefined threshold (47% in pregnant vs. 43% in non-pregnant participants), reflecting systematic bias between methods. In pregnant participants, the mean difference (rectal probe - CRS) was −0.23°C (95% CI: −0.36, −0.09°C), indicating that CRS overestimated Tcore relative to the rectal probe. In contrast, in non-pregnant participants, the mean difference was 0.16°C (95% CI: 0.03, 0.28°C), indicating that CRS underestimated Tcore. Discrepancies were most pronounced during the early intervals but diminished over time.

**Conclusion:**

This study shows opposing systematic bias of CRS in pregnant and non-pregnant women compared with Tcore measured using a rectal probe, limiting its use as a stand-alone tool in pregnancy and highlighting the need for non-invasive temperature sensor development.

## 1 Introduction

Physical activity during pregnancy is widely recommended due to its numerous health benefits for both the mother and fetus.^1^, ^2^, ^3^, ^4^ While physical activity guidelines recommend at least 150 min of moderate-intensity physical activity per week during pregnancy,^5^, ^6^, ^7^, ^8^, ^9^ some elite and recreational athletes may wish to continue structured training, including high-intensity exercise, during pregnancy.^5^^,^^10^, ^11^, ^12^ Such training may extend into the second and third trimester, although the duration and intensity of continued training are highly individual and depend on the course of pregnancy, maternal symptoms, training background, and medical advice. This type of exercise can increase core temperature (Tcore),^13^^,^^14^ raising concerns about maternal hyperthermia (>39°C) and its potential risks reported in the literature include neural tube defects^15^ during early pregnancy, as well as preterm birth^16^ and fetal growth restriction^16^ later in pregnancy. As the potential fetal risk associated with maternal hyperthermia is considered particularly relevant during the first trimester, when organogenesis occurs,^15^ it is important to distinguish between early and late pregnancy. The present study included pregnant athletes from gestational week 26 and therefore reflects responses in late pregnancy. Several methods exist for measuring Tcore during exercise, but few offer both ease of use and measurement precision.^17^, ^18^, ^19^, ^20^, ^21^, ^22^, ^23^ Accurate assessment of Tcore is essential when evaluating heat strain, yet commonly used invasive methods, including rectal, esophageal, pulmonary artery, and urinary bladder measurements, require medical expertise and may pose hygiene and feasibility challenges. Core temperature measured using a rectal probe is most commonly used^24^, ^25^, ^26^ and regarded as the gold standard for Tcore measurement in exercising individuals and hyperthermic athletes^23^^,^^27^^,^^28^ and recommended by the National Athletic Trainers Association.^29^ Despite being the reference method in research, a rectal probe is invasive and impractical for repeated or continuous measurements during exercise due to discomfort and interruption of activity. Ingestible telemetric pills are widely used in exercise research.^20^^,^^30^, ^31^, ^32^, ^33^, ^34^, ^35^, ^36^, ^37^ However, they are costly and the manufacturer lists pregnancy as a contraindication.^38^ The reason for this contraindication is not specified, but it may reflect the limited safety data available for pregnant women and fetuses. Consequently, there is a clear need for non-invasive methods that provide accurate Tcore estimates without introducing potential risks during pregnancy.

The CALERA Research Sensor (CRS) (greenTEG AG, Switzerland) is a research-grade version of the commercially available CORE sensor, based on the same underlying technology but with features relevant for research use, including 1-s data resolution and access to raw data.^33^^,^^39^^,^^40^ The non-invasive CRS determines Tcore by integrating measurements of skin temperature, heat flux, and heart rate, utilizing machine learning algorithms. An external validation study reported biases of 0.23°C and −0.10°C compared with rectal temperature during exercise.^41^

Although CRS has been used in four exercise and sport studies,^32^^,^^33^^,^^42^^,^^43^ only three have evaluated its validity against a reference method for Tcore during exercise,^32^^,^^33^^,^^43^ and only two of these were conducted in healthy individuals.^32^^,^^43^ However, its accuracy has not yet been examined in pregnant women, where precise and safe temperature monitoring is particularly important given the risk of maternal hyperthermia. The present study addresses this gap by evaluating the performance of CRS in pregnant women, to ensure accurate temperature assessment and support safe exercise recommendations during pregnancy. This is particularly relevant because the CRS estimates Tcore from skin temperature and heart rate, which may be influenced by pregnancy-related adaptations such as increased blood volume, cardiac output, resting heart rate, skin blood flow, heat dissipation, and metabolic heat production.^10^^,^^14^^,^^44^^,^^45^ In the same study population, pregnant participants have previously shown a greater increase in skin temperature following high-intensity running compared with non-pregnant controls (4.08 ± 0.72°C vs. 3.25 ± 0.86°C, p = 0.008).^14^ Therefore, validity demonstrated in non-pregnant populations cannot necessarily be assumed to apply to pregnant women. Considering these pregnancy-related thermoregulatory and cardiovascular adaptations, the aim of this study was to assess agreement and systematic differences between Tcore measured with CRS and a rectal probe during high-intensity interval running in elite and recreational athletes, both pregnant and non-pregnant.

## 2 Materials and methods

### 2.1 Design and participants

This study was a part of the “Strong Mama project”.^14^^,^^46^, ^47^, ^48^ Data collection took place from September 2022 to May 2023. Due to limited research on pregnant women, an accurate power calculation was not feasible. A total sample size of 30 participants was therefore selected, including 15 pregnant and 15 non-pregnant women. This sample size exceeded those used in previous Tcore sensor validation studies conducted in non-pregnant populations.^31^, ^32^, ^33^^,^^41^ In addition, the non-pregnant group exceeded the minimum sample size of eight participants estimated by Dannen et al. in a comparable validation study.^49^ Pregnant elite and recreational athletes were recruited from the Strong Mama project, while non-pregnant participants were recruited through fitness clubs, social media, and students at the Norwegian School of Sport Sciences (NIH). Pregnant participants were assessed from gestational week 26, in line with the Strong Mama protocol and considerations regarding fetal well-being measures in later pregnancy.^50^ All participants were informed about the study's purpose, procedures, and use of temperature-monitoring devices. Elite status was defined as current membership in a national team or equivalent high-level representative team recognized by a National Sports Federation. Elite and recreational athletes were analyzed as a single group because all participants met comparable training criteria and followed the same standardized exercise protocol. All participants reported a minimum of 4 h of structured endurance or strength training per week, including regular high-intensity sessions, for at least two years before pregnancy (or study enrolment for non-pregnant participants). Participants were recruited as part of the Strong Mama project, from which related outcomes have been reported previously,^14^^,^^46^, ^47^, ^48^ all methodological details relevant to the present analysis are provided below. Non-pregnant participants were required to use combined hormonal contraceptives (oral contraceptive pills, contraceptive patches or vaginal rings) and were tested during active hormone use, not during the withdrawal bleeding phase, to minimize hormonal variability and potential effects on thermoregulation. Background data included pre-pregnancy weight and training history. Pregnant participants were healthy athletes accustomed to high-intensity exercise, and individuals with medical obstetric contraindications to exercise^51^ were excluded. Several safety procedures were implemented during the exercise testing session, including the presence of a medical doctor trained in maternal–fetal medicine, ultrasound monitoring of fetal responses during the rest periods between intervals, predefined stopping criteria, and a protocol for referral to hospital for further follow-up in case of abnormal findings.

### 2.2 Measures

#### 2.2.1 Rectal probe

A rectal probe (Gemini Data Loggers Ltd, PB-5015-1M5, Chichester, UK; 1.5m long, 6.5 mm cable diameter, 150g) (Fig. 1), served as the reference method and was calibrated by an ISO-certified laboratory, with an expanded uncertainty of ±0.109°C. The probe was equipped with a small stabilizer positioned 10 cm from the probe tip to maintain a consistent depth. Following standardized instructions from the researcher, participants self-inserted the probe approximately 10 cm past the anal sphincter.^52^^,^^53^ Core temperature was measured continuously at 1-s intervals using a logging device with an integral display (Gemini Data Loggers Ltd, TinyTag® View 2, TV-4020, West Sussex, UK). Data was stored on the logging device and later transferred to a computer using a USB download cable and EasyView Pro software (version 5.6). All temperature data were saved as text document (.txt), imported into Microsoft Excel and stored as Excel files (.xlsx).

**Fig. 1:**
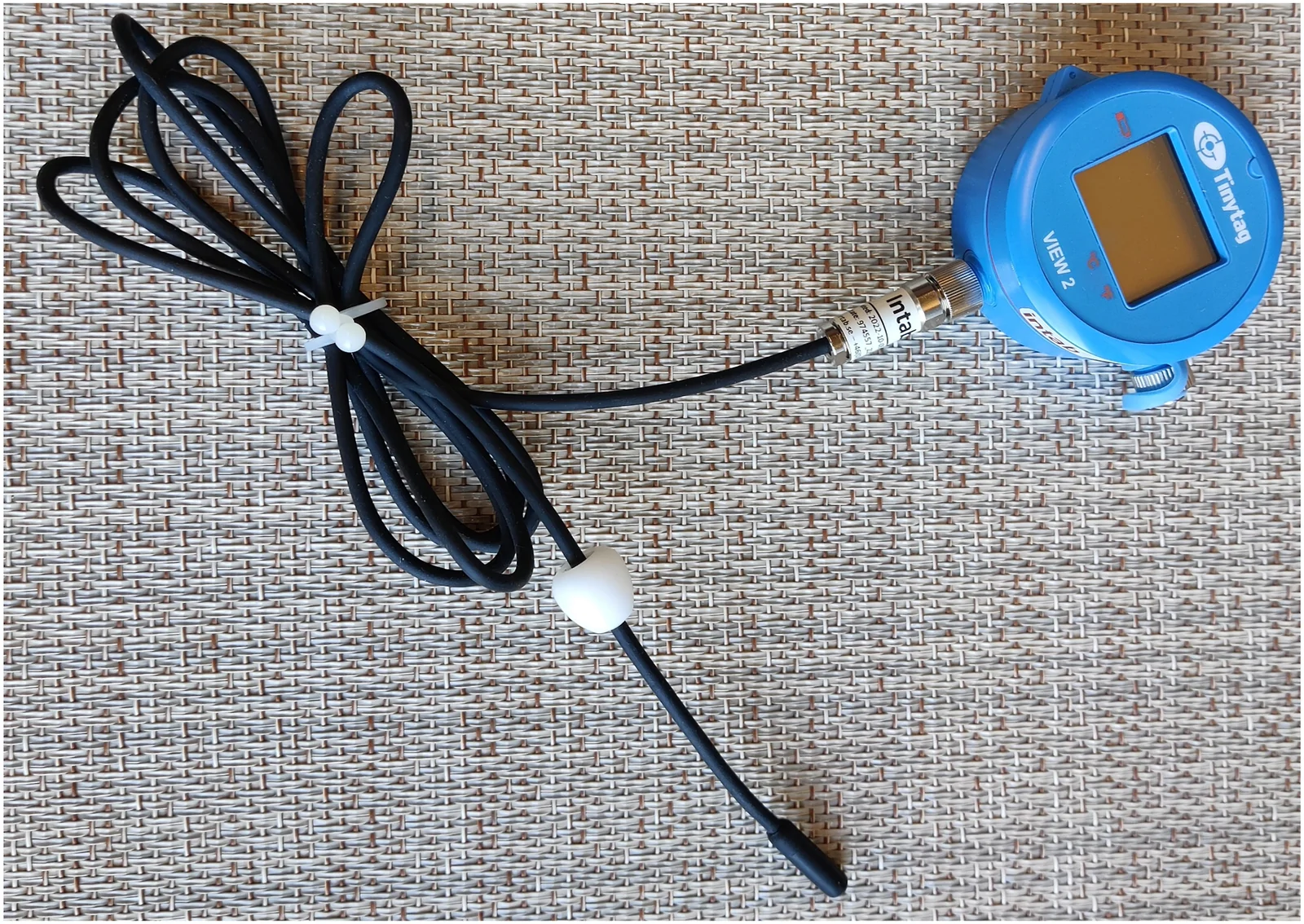
The rectal probe and the Tinytag View2. (Photo by Sofia Brevik-Persson)^14^ Reproduced from Brevik-Persson et al. (2024), Journal of Exercise Science & Fitness, under CC BY 4.0 (https://creativecommons.org/licenses/by/4.0/). https://doi.org/10.1016/j.jesf.2024.09.003.

#### 2.2.2 CALERA Research Sensor

Core temperature was measured non-invasively using CRS (50x40 × 8.35 mm, 17g). All CRS underwent individualized factory calibration at the manufacturer's headquarters prior to shipping, and no manual participant-specific calibration was required, as present study allowed all participants a 20-min stabilization period after applying the sensor before data were used. The CALERA Research Sensor was attached to a heart rate belt and positioned on the left apical chest (Fig. 2). Following the manufacturer ꞌs instructions for exercise-specific algorithms, the sensor was paired with a heart rate monitor with an ANT + connection (Polar H10). Core temperature was recorded every second and stored during measurement and transferred to a computer using a USB-receiver with Bluetooth connection to Core Body Temperature Research Tool software (firmware version 0.6.2). All stored data were downloaded from the software and saved as a CSV file for further analysis.

**Fig. 2:**
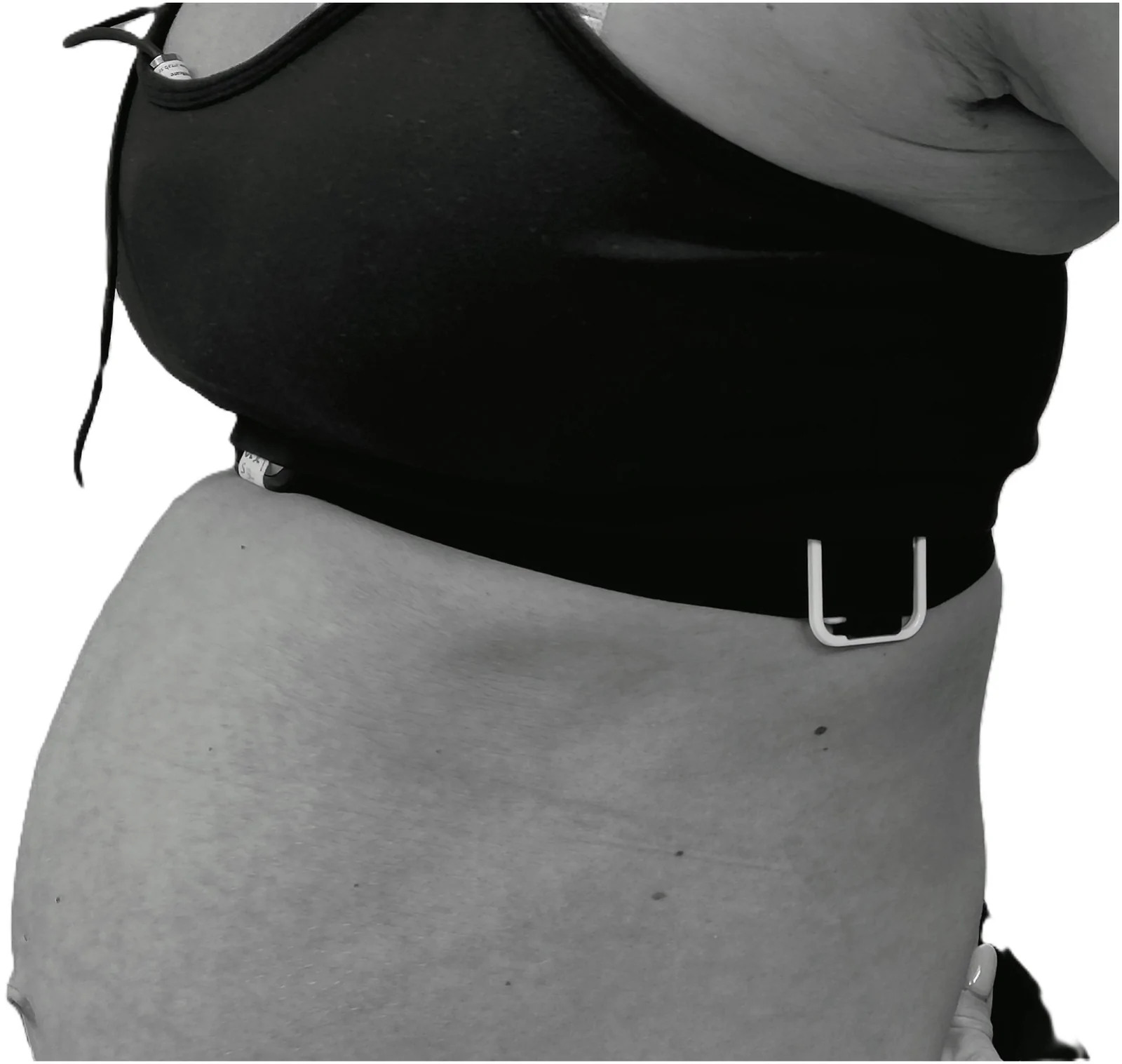
The CALERA Research Sensor attached to a heart rate belt and positioned at the left apical. (Photo by Sofia Brevik-Persson).

### 2.3 Study protocol

The protocol was first piloted in a small group to ensure feasibility and safety. Prior to the interval sessions, participants were instructed to avoid food and drink at least 2 h and were contacted in advance to confirm adherence.^14^

#### 2.3.1 Exercise protocol

The treadmill protocol consisted of a 10-min warmup, up to five 5-min treadmill intervals, and a 4-min rest periods between intervals. The treadmill protocol began with a 10-min warm-up at gradually increasing intensity. Participants completed up to five 5-min treadmill-intervals (Woodway, Weil am Rhein, Germany) at a target intensity of 17 RPE or 90% of maximal heart rate.^14^^,^^47^ Pace was self-selected during the first interval and adjusted to maintain the target intensity. The treadmill incline remained constant at 3°. Each interval was followed by a 4-min rest period. During the rest periods, pregnant participants underwent ultrasound monitoring to assess fetal responses and to support safety monitoring during the test, while non-pregnant participants remained seated. Ultrasound monitoring was performed using a GE Healthcare Voluson E8/E10/E22 Doppler Ultrasound with XDclear Probe (Oslo, Norway). During ultrasound assessment, gel was applied locally to the maternal abdomen according to standard procedures, while the CRS was positioned on the thorax, separate from the ultrasound examination area. The protocol included stopping criteria such as abnormal fetal heart rate (<110 bpm >3-min, >180 bpm >6-min), low oxygen saturation, or discomfort. Participants could withdraw at any time.

#### 2.3.2 Temperature recording and environmental conditions

Baseline temperatures were measured before the warmup, with minute 0 marking the start of the first interval. Temperature was recorded from 20-min before to 20-min after interval running. All measurements were conducted in a controlled laboratory setting at NIH, Oslo, supervised by two clinical exercise physiologists. Environmental conditions (temperature: 20°C to 21°C; relative humidity, 38% to 61%) were continuously monitored and recorded (Nielsen-Kellerman Company, Kestrel 5400 Heat Stress Tracker, Pennsylvania, USA), to ensure consistency.

### 2.4 Ethical approval

The study was approved by the Regional Committee for Medical and Health Research Ethics, Southern Norway, Oslo (REK 478976), and the Norwegian Social Science Service (Sikt 628051).14 All participants provided informed consent in accordance with the Helsinki Declaration.49 Participation was voluntary and data was non-identifiable and handled confidentially. The study was funded by The Norwegian Women's Public Health Association. No participant compensation was provided. Data were stored at NIH in line with Norwegian regulations and will be retained for a minimum of five years following the study completion.

### 2.5 Statistical analysis

Statistical analyses and graphical outputs were performed using R statistical software (version 4.6.0). Data from one pregnant participant (baseline to first interval) were excluded due to improper rectal probe insertion. Additionally, data from one non-pregnant participant were excluded because of rectal probe measurement errors. Normality was assessed with the Shapiro-Wilk test (p > 0.05). A pre-defined ±0.3°C acceptability threshold was applied, consistent with previous validation studies.^31^^,^^33^^,^^41^ Agreement between the rectal probe and CRS was evaluated using Bland–Altman plots based on per-minute data. The predefined ±0.3°C acceptability threshold was visualized in the plots. Mean bias was calculated as the mean paired difference between rectal probe and CRS measurements, and the 95% limits of agreement were calculated as the mean bias ± 1.96 SD of the paired differences based on individual mean values. The proportion of paired measurements falling within the threshold of ±0.3°C was calculated directly from the observed data and is presented as descriptive statistics.

Data are presented as mean ± standard deviation (SD), and 95% CI. Between-group differences in participant characteristics presented in Table 1 were analyzed using independent-samples t-tests. Differences between rectal probe and CRS measurements were analyzed using paired *t*-test based on average individual data. For interval-wise comparisons across the exercise protocol, the familywise error rate was controlled using the Holm method.^54^

**Table 1:** Characteristics of pregnant and non-pregnant participants included in the present analysis. Participants characteristics for the original population have been reported previously by Brevik-Persson et al. (2024),^14^ Journal of Exercise Science & Fitness, under CC BY 4.0 (https://creativecommons.org/licenses/by/4.0/). https://doi.org/10.1016/j.jesf.2024.09.003 Values in the present table were recalculated for the current validation analysis.

Characteristics	Group			
	Pregnant		Non-pregnant	
	n	Mean ± SD	n	Mean ± SD
Age (y)	15	31 ± 4	14	29 ± 5
Gestational age (weeks)	15	29.8 ± 2.9	-	-
BMI (kg/m^2^)	14	21.9 ± 1.7^a^^,^^b^	14	23.7 ± 2.1
Weight (kg)	15	72.2 ± 6.4	14	65.8 ± 5.6
GWG^b^	14	10.8 ± 2.4	-	-
Height (cm)	15	168.0 ± 5.7	14	167.8 ± 5.8
Exercise frequency (minutes/wk)^b^	14	342 ± 116	14	296 ± 50
Baseline Core temp. (°C)^c^	15	37.0 ± 0.2	14	37.5 ± 0.3*
Baseline skin temp. (°C)^c^	15	31.4 ± 0.8	14	30.2 ± 0.5*
Estimated maximal heart rate^d^	15	197.0 ± 2.2	14	191.9 ± 7.9

BMI, body mass index = weight/(height)^2^; GWG, gestational weight gain; SD, Standard Deviation.

*Significant differences between pregnant and non-pregnant participants (independent-samples *t*-test; p < 0.05).

a Pre-pregnancy BMI (kg/m^2^).

b Self-reported data.

c Baseline core temperature (measured with a rectal probe)- and skin temperature were measured before the warm-up.

d Estimated maximal heart rate among the pregnant participants was calculated as earlier described by Mottola et al. [55], and among the non-pregnant participants 220-age if the participants were unaware of their own maximal heart rate.

As a sensitivity analysis to address incomplete interval data in the pregnant group, a linear mixed-effects model (LMM) was fitted using the lmerTest package in R (version 4.6.0). Because missing data in the later intervals were related to predefined stopping criteria rather than random participant withdrawal, the analysis was used to assess whether this non-random missingness influenced the findings. The model included sensor (rectal thermometer vs. CALERA Research Sensor), group (pregnant vs. non-pregnant), exercise phase (baseline and intervals 1–5), and all pairwise interactions as fixed effects, with a random intercept for participant to account for repeated measures. Estimated marginal means and sensor contrasts were derived using the emmeans package with Bonferroni adjustment.

Although per-second measurements are temporally autocorrelated, this approach was considered appropriate for detecting systematic differences between methods, as both sensors recorded synchronously and were exposed to identical physiological conditions. The significance level was set at p < 0.05.

## 3 Results

### 3.1 Participant characteristics

Groups were comparable (Table 1), each including one elite athlete and 14 recreational athletes. All participants were of Nordic Caucasian origin. Gestational age among pregnant participants ranged from 25 + 6 to 35 + 2 weeks. Among the pregnant participants, four completed all five exercise intervals. Earlier termination of the protocol occurred due to participant preference (n = 3), abnormal fetal heart rate (n = 4), or exceeding 90% of estimated maximal heart rate (n = 4), in accordance with predefined stopping criteria. All the participants in the non-pregnant group (n = 15) completed all intervals.

### 3.2 Agreement analysis

A substantial proportion of CRS measurements exceeded the ±0.3°C acceptability threshold (47% in pregnant and 43% in non-pregnant group, Table 2). Bland-Altman plots demonstrate the direction and magnitude of mean bias between methods (Fig. 3). Mean Tcore, method differences and both unadjusted and Holm-adjusted p-values are presented in Table 3. In the pregnant group, CRS showed higher values than the rectal probe (Fig. 4). Significant unadjusted differences were observed at baseline and during interval one to three, however, after Holm adjustment, the baseline comparison and interval three were no longer statistically significant. In the non-pregnant group, CRS recorded lower values than the rectal probe, with significant differences at baseline and interval one. The total exercise comparison was significant before, but not after, Holm adjustment.

**Table 2:** Agreement between the rectal probe and the CALERA Research Sensor in pregnant and non-pregnant women.

	Mean Difference [C°]	95% CI of the Difference [C°]		*p*-value	LoA (d±1.96SD) [C°]		Within ±0.3°C^a^ [%]
		Lower	Upper		Lower	Upper	
Pregnant	−0.23	−0.36	−0.09	0.0026	−0.70	0.24	53
Non-pregnant	0.16	0.03	0.28	0.0160	−0.26	0.58	57

CI, Confidence Interval; SD, Standard deviation; LoA, Limit of Agreement.

Bias was calculated as rectal temperature minus CALERA Research Sensor temperature; negative values indicate higher CALERA readings and positive values indicate lower CALERA readings relative to the rectal probe.

a The percentage of paired measurements within the predefined acceptable threshold of ±0.3°C is presented as descriptive statistics, calculated directly from the observed per second data.

**Fig. 3:**
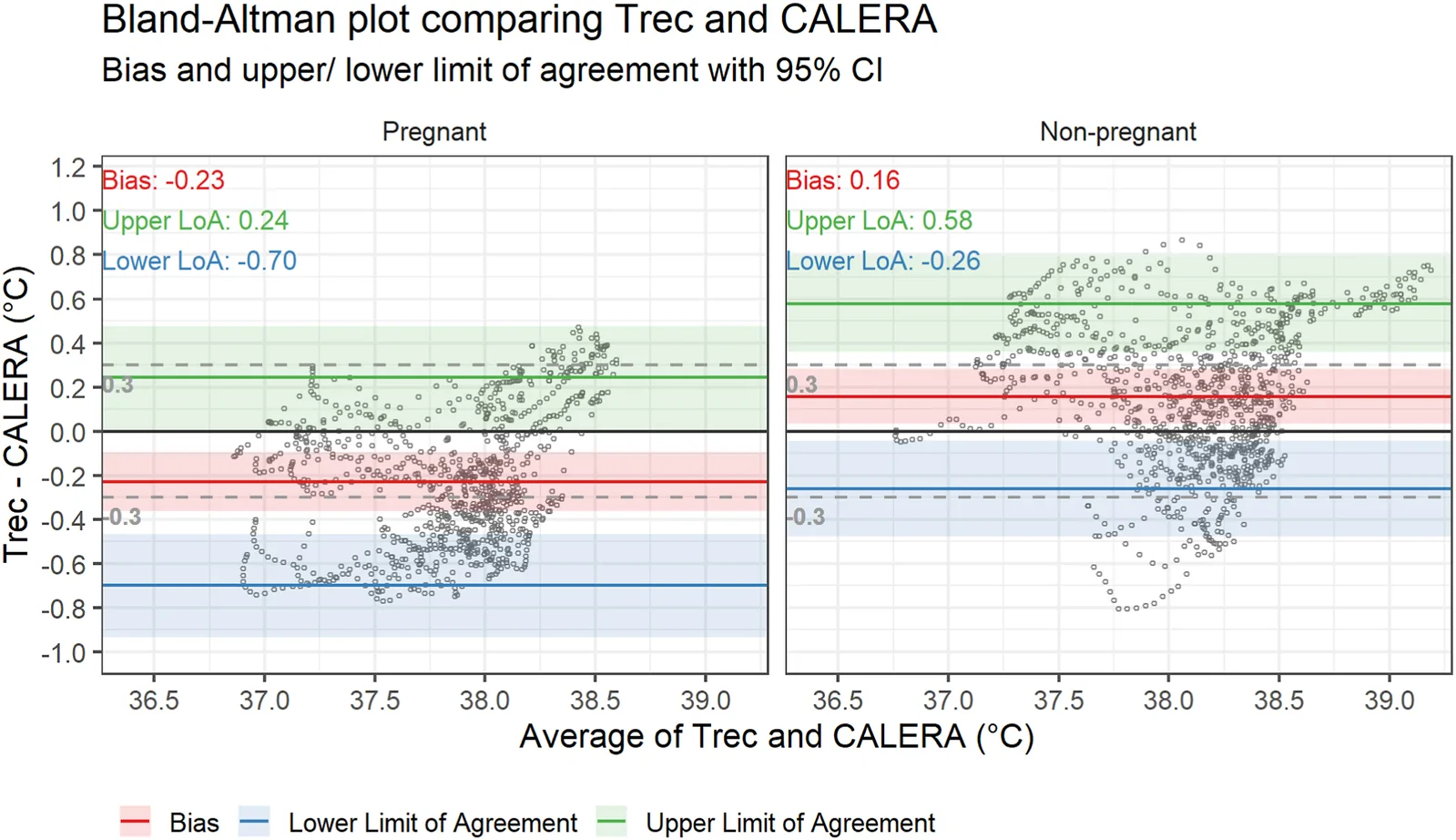
Bland-Altman plot comparing rectal probe (Trec) and CALERA Research Sensor in the pregnant group and the non-pregnant group. Data once per minute from warm-up to 20 min post-exercise. The Y-axis shows the mean difference between the methods, calculated as Trec minus CALERA Research Sensor. Solid lines represent the mean bias and the upper and lower 95% confidence intervals. Dashed lines represent the pre-defined acceptable threshold (±0.3°C).

**Table 3:** Mean core temperature (˚C) measured per second by the rectal probe (Trec) and the CALERA Research Sensor from warm-up to 20 min post-exercise, as well as during each interval, for both pregnant (a) and non-pregnant (b) groups.

Pregnant							
	n	Trec [°C]	CALERA [°C]	Difference [°C]	95% CI	*p*-value	Adjusted^a^
		Mean ± SD	Mean ± SD	Mean ± SD			
Baseline	15	37.01 ± 0.21	37.16 ± 0.13	−0.14 ± 0.23^c^	−0.27, −0.02	0.03	0.19

Total exercise	14	37.73 ± 0.26	37.96 ± 0.15	−0.23 ± 0.24 c	−0.36, −0.09	0.00	0.03

Interval							

1	15	37.51 ± 0.25	37.77 ± 0.13	−0.27 ± 0.26^c^	−0.41, −0.12	0.00	0.01
2	15	37.77 ± 0.27	38.09 ± 0.13	−0.32 ± 0.27^c^	−0.47, −0.17	0.00	0.00
3	13^b^	37.92 ± 0.31	38.18 ± 0.11	−0.26 ± 0.30^c^	−0.44, −0.08	0.01	0.08
4	8^b^	38.16 ± 0.33	38.24 ± 0.12	−0.08 ± 0.29^c^	−0.32, −0.16	0.46	0.92
5	4^b^	38.26 ± 0.38	38.35 ± 0.10	−0.09 ± 0.36^c^	−0.65, −0.48	0.66	0.92

Non-pregnant							
	n	Trec [°C]	CALERA [°C]	Difference [°C]	95% CI	*p*-value	Adjusted^a^
		Mean ± SD	Mean ± SD	Mean ± SD			
Baseline	14	37.48 ± 0.27	37.06 ± 0.13	0.42 ± 0.20^c^	0.30, 0.53	0.00	0.00

Total exercise	14	38.18 ± 0.30	38.02 ± 0.16	0.16 ± 0.21^c^	0.03, 0.28	0.02	0.13

Interval							

1	14	38.01 ± 0.34	37.71 ± 0.22	0.31 ± 0.23^c^	0.18, 0.44	0.00	0.00
2	14	38.17 ± 0.33	38.07 ± 0.19	0.10 ± 0.22^c^	−0.02, 0.23	0.10	0.55
3	14	38.33 ± 0.32	38.22 ± 0.19	0.11 ± 0.24^c^	−0.03, 0.24	0.12	0.55
4	14	38.42 ± 0.30	38.29 ± 0.18	0.13 ± 0.26^c^	−0.02, 0.28	0.09	0.55
5	14	38.47 ± 0.34	38.36 ± 0.16	0.11 ± 0.30^c^	−0.07-0.28	0.21	0.62

CI, Confidence Interval; SD, Standard Deviation.

a Adjusted using Holm method: [Holm S (1979): https://www.jstor.org/stable/4615733].

b n decreases due to stopping criteria described in methods.

c Sensor differences are calculated as core temperature measured with the rectal probe (Trec) minus CALERA Research Sensor measurements; positive values indicate higher core temperature measured using the rectal probe relative to the CALERA Research Sensor.

**Fig. 4:**
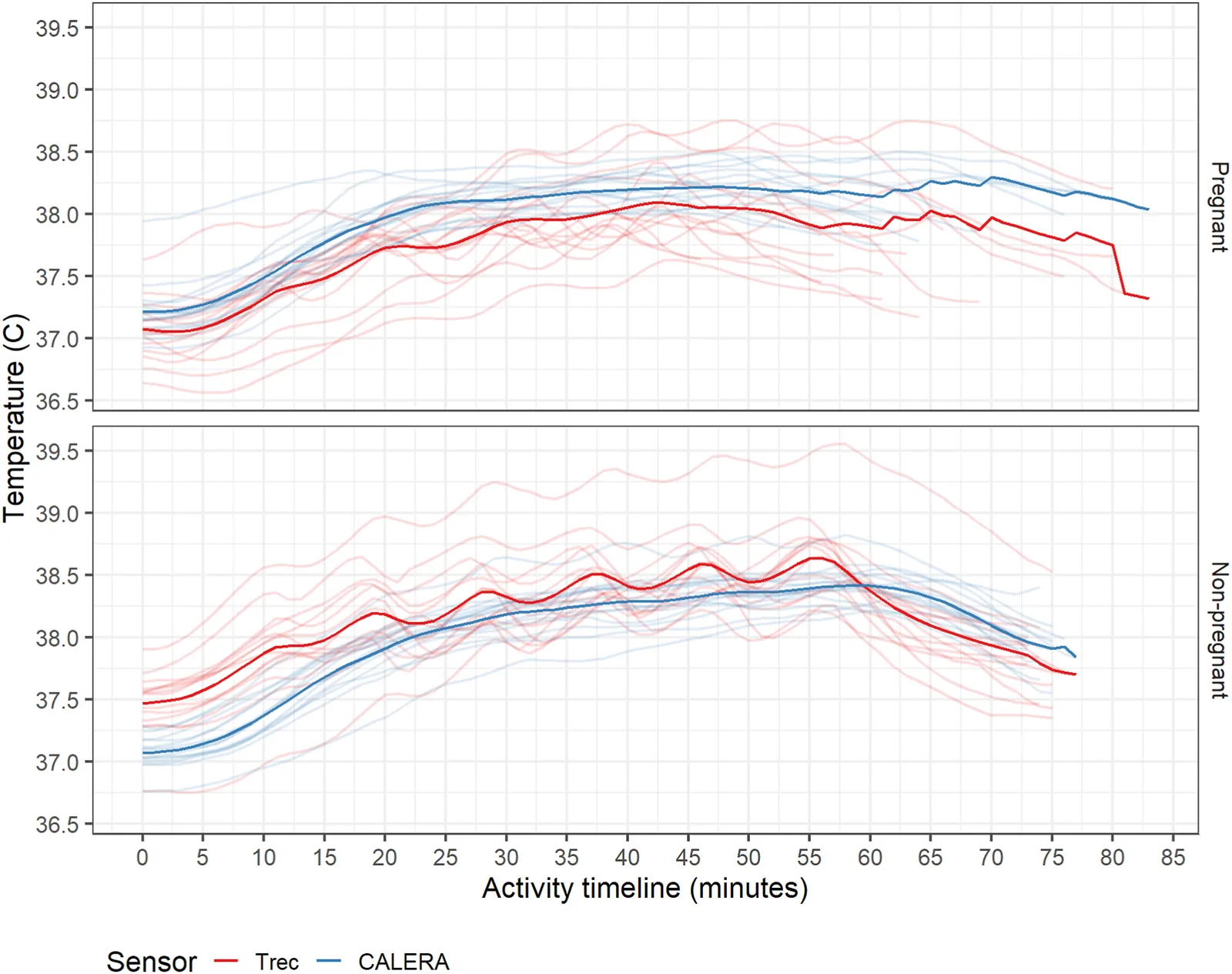
Individual temperature trajectories measured by the rectal probe (Trec) and the CALERA Research Sensor in pregnant and non-pregnant women from interval 1 to 20 min post-exercise. Thin lines indicate individual participants, and the thicker line indicates the group mean.

As a sensitivity analysis, a linear mixed-effects model fitted on all available per-minute data confirmed the overall pattern of sensor bias observed in the primary analyses, with CRS overestimating Trec in the pregnant group and underestimating Trec in the non-pregnant group across the exercise protocol. To formally assess whether incomplete interval data in the pregnant group affected these findings, a joint model including a binary indicator for completion of all five intervals was fitted. Four pregnant participants and 14 non-pregnant participants included in the final analyses completed all five intervals. The sensor × completion status interaction was statistically significant (F(1, 4318) = 9.32, p = 0.002), indicating that the magnitude of bias differed between participants who completed all intervals and those who ended the protocol earlier. Importantly, the direction of bias remained consistent across the full-sample and completed-interval models.

## 4 Discussion

In line with the study aim of assessing agreement and systematic differences between a non-invasive wearable sensor and a rectal probe, this study evaluated the accuracy of CRS for estimating Tcore during high-intensity interval running. Agreement was limited in both pregnant and non-pregnant participants, with only 53% and 57% of readings within the predefined ±0.3°C threshold, respectively. The most important finding was the opposing direction of systematic bias between groups. CALERA Research Sensor overestimated Tcore in pregnant participants but underestimated it in non-pregnant participants, suggesting limited validity during high-intensity intervals.

To our knowledge, this is the first study assessing CRS accuracy in pregnancy. The overestimation observed in pregnant participants may reflect pregnancy-related cardiovascular and thermoregulatory adaptions, including altered heart rate responses, increased skin blood flow, and enhanced peripheral heat dissipation. These adaptations influence the variables used by the CRS algorithm and thereby may affect agreement with rectal temperature. In the present study, raw torso skin temperature was not measured independently. Therefore, the specific contribution of local skin temperature, including any potential influence of sensor placement close to the uterus, cannot be determined. Nevertheless, previously reported findings from the same study population,^14^ including lower Tcore and greater skin temperature responses during exercise in pregnancy, support the possibility that altered peripheral heat dissipation contributed to the systematic overestimation observed in pregnant participants. This interpretation should be made cautiously and requires confirmation in future studies.

In contrast, CRS underestimated Tcore in the non-pregnant group. This finding suggests that the direction and magnitude of CRS bias may depend not only on the sensor technology itself, but also on physiological state and exercise context. The inclusion of both pregnant and non-pregnant women therefore provides additional insight into how CRS behavior may differ between populations exposed to the same exercise protocol.

Previous CRS validation studies in healthy individuals used ingestible telemetric pills as the reference method during cycling exercise and reported variable findings, including high agreement in Januário et al.^32^ and underestimation of Tcore in Priego-Quesada et al.^43^ However, ingestible telemetric pills may be influenced by gastrointestinal transit time and pill location, whereas rectal temperature is commonly used as a laboratory reference measure of Tcore.^18^^,^^23^^,^^27^^,^^28^ Direct comparison with the present study is also limited by differences in agreement criteria, data frequency, firmware version, exercise protocol, and study population. Because CRS-specific validation evidence remains limited, findings from studies evaluating the commercially available CORE sensor may provide contextual background, although they should be interpreted cautiously. CORE and CRS are both developed by greenTEG AG (Switzerland) and are based on related measurement principles, but they are not identical devices. Previous CORE studies have reported both systematic overestimation of Tcore, consistent with the bias observed in the pregnant participants in the present study,^41^^,^^49^ and systematic underestimation, more consistent with findings in our non-pregnant group.^31^^,^^33^^,^^56^ Taken together, the available evidence suggests that agreement between wearable sensors and reference measures is context-dependent and may vary across populations, exercise conditions, reference methods and sensor algorithms.

Given the limited agreement observed across groups, calibration should also be considered as a potential explanation. The CRS sensors used in the present study underwent individualized factory calibration by the manufacturer, and no manual participant-specific calibration was required.^39^^,^^40^ All participants completed at least a 20-min stabilization period after CRS application before the running protocol started, in accordance with manufacturer recommendations. The rectal probes were also individually factory-calibrated. Therefore, insufficient calibration is unlikely to be the primary explanation for the limited agreement between CRS-derived and rectal probe measurements, although the use of the same CRS algorithm across participants may still have contributed to the observed discrepancies.

### 4.1 Sensor equilibration

Based on the available interval data, discrepancies between CRS and the rectal probe were most pronounced during the early exercise intervals and diminished over time. This temporal pattern may indicate that CRS require a prolonged equilibration period before providing stable estimates during high-intensity interval exercise. However, this interpretation should be made cautiously, particularly for the pregnant group, as fewer participants completed the later intervals. In addition, rapid changes in Tcore during interval exercise may increase discrepancies between measurement sites because different Tcore methods differ in response time. For example, rectal temperature has been shown to respond more slowly than esophageal temperature during dynamic exercise.^57^

### 4.2 Practical implications

Given the relatively small sample size and the unequal completion of intervals, particularly in the pregnant group, these practical implications should be interpreted cautiously. The findings suggest that CRS measurements during high-intensity interval exercise should not be interpreted as direct substitutes for reference measures of Tcore. In pregnant women, the observed overestimation indicated that the sensor should not currently be used as a stand-alone tool to assess heat strain during exercise. In non-pregnant athletes, the observed underestimation suggests that absolute CRS measurements may underestimate true thermal load during high-intensity interval exercise.

Overall, CRS may provide a convenient, non-invasive option for monitoring relative within-session temperature trends, but further algorithm development, particularly accounting for pregnancy-related physiological adaptations, are needed before the device can be recommended for independent and accurately measuring absolute core temperature.

### 4.3 Strength and limitations

A key strength of the present study is the inclusion of both pregnant and non-pregnant women within the same experimental design, allowing direct comparison of wearable sensor performance across distinct physiological states. This approach enabled identification of pregnancy-specific differences in systematic bias, a perspective not previously addressed in validation studies of non-invasive Tcore sensors. A further strength is the use of a rectal probe as the reference method, providing a robust gold-standard comparison in a population where accurate temperature monitoring is particularly critical. The exercise protocol was standardized with respect to predefined exercise intensity targets, fixed interval and rest durations, and stable environmental conditions. This standardization refers to the testing conditions and structure of the completed intervals, while unequal interval completion is addressed as an important limitation below. Furthermore, the total sample size was larger than previous validation studies of non-invasive Tcore sensors, strengthening the robustness and interpretability of the findings.

Despite these strengths, several limitations should be acknowledged. An important limitation concerns the unequal completion of intervals between groups. Only four of the fifteen pregnant participants completed all five exercise intervals, as participants were withdrawn according to predefined stopping criteria, including fetal heart rate changes and maternal heart rate exceeding 90% of HRmax. Consequently, findings from the later exercise intervals are based on a small, physiologically selected subgroup and should therefore be interpreted with caution rather than generalized to the broader population of pregnant women. In addition, the self-paced components of the exercise protocol may have resulted in some differences in total work completed between groups. However, agreement between temperature measurement methods was evaluated within participants at corresponding exercise phases rather than by comparing physiological responses between groups. The findings are also specific to firmware version 0.6.2 of CRS, and future algorithm updates may alter accuracy. The study was limited to treadmill-based high-intensity intervals in elite and recreational athletes, and results may therefore not be directly generalizable across other exercise modalities, environmental conditions, or populations. Finally, although pregnant participants underwent routine ultrasound assessment during rest periods, the CRS was positioned separately from the ultrasound examination area, and no gel was applied in proximity to the sensor. Any local thermal effect of the gel was therefore unlikely to influence CRS- or rectal Tcore measurements.

Future research could combine CRS measurements with thermal imaging of the torso in both pregnant and non-pregnant participants to provide greater insight into local skin temperature distribution and clarify whether pregnancy-related differences in peripheral heat dissipation contribute to algorithmic bias. In addition, future studies including naturally cycling non-pregnant participants may benefit from testing participants during the follicular phase, when Tcore is generally more stable.

## 5 Conclusions

This study shows that the CALERA Research Sensor exhibits opposing systematic bias in pregnant and non-pregnant women compared with Tcore measured using a rectal probe. This limits the suitability of the sensor as a stand-alone tool for monitoring heat strain in pregnancy, highlighting the need for further development of non-invasive Tcore sensors that account for physiological state, particularly in pregnant populations.

This project is funded by the Norwegian women's Public Health Association.

## Data availability statement

The datasets used and analyzed in this study are available from the first author on reasonable request.

## Author contributions

LAHH is the main supervisor and initiative taker for the Strong Mama project and has served as mentor for the present study together with CG. SBP has outlined the present manuscript and been responsible for data collection together with EMD. JM has provided his academic expertise in the field of temperature, as well contributed to data interpretation, and provided critical feedback together with LAHH, CG and EMD. All authors reviewed and approved the final manuscript, and the publication has been approved by the responsible authorities at the institution where the work has been carried out.

## Declaration of interests

The authors declare that they have no known competing financial interests or personal relationships that could have appeared to influence the work reported in this paper.
